# Interplay Between KLF4, STAT, IRF, and NF-κB in VSMC and Macrophage Plasticity During Vascular Inflammation and Atherosclerosis

**DOI:** 10.3390/ijms262010205

**Published:** 2025-10-20

**Authors:** Natalia Lopacinska, Joanna Wesoly, Hans A. R. Bluyssen

**Affiliations:** 1 Human Molecular Genetics Research Unit, Institute of Molecular Biology and Biotechnology, Faculty of Biology, Adam Mickiewicz University, 61-614 Poznan, Poland; 2 Laboratory of High Throughput Technologies, Faculty of Biology, Adam Mickiewicz University, 61-614 Poznan, Poland

**Keywords:** atherosclerosis, Vascular Smooth Muscle Cell (VSMC) and macrophage plasticity, Interferon gamma (IFNγ) and Toll-like receptor (TLR)4 signaling, Krüppel-like factor 4 (KLF4), Signal Transducer and Activator of Transcription (STAT), Interferon Regulatory Factor (IRF), Nuclear factor-κB (NF-κB), transcriptional networks

## Abstract

Atherosclerosis is characterized by atherosclerotic plaque formation in large and medium vessels, mediated by endothelial cell (EC) dysfunction, altered contractility of Vascular Smooth Muscle Cells (VSMCs) and recruitment of blood leukocytes to the injured vascular endothelium. These include macrophages (MØ), T lymphocytes, and dendritic cells, which drive the production of many inflammatory mediators and the process of chronic inflammation. Also, de-differentiation or phenotypic switching of VSMCs contributes to vascular remodeling and the pathogenesis of atherosclerosis. Likewise, MØ plasticity and the presence of different phenotypes have a major effect on atherosclerotic plaque formation. The multi-functional transcriptional regulator and pluripotency factor Krüppel-like factor 4 (KLF4) acts as a gatekeeper of VSMC phenotypic switching and MØ polarization during vascular inflammation and atherosclerosis. Similarly, pro-inflammatory pathways activated by Toll-like receptor (TLR)4 and Interferon gamma (IFNγ) emerge as key components of VSMC and MØ plasticity, tightly regulated by Signal Transducer and Activator of Transcription (STAT)s, Interferon Regulatory Factor (IRF)s, and Nuclear factor-κB (NF-κB). Recent discoveries predict a collaborative role of these transcription factors in different transcriptional mechanisms connected to inflammation and atherosclerosis. This review provides novel insight into the transcriptional regulatory interplay between KLF4, STATs, IRFs, and NF-κB in VSMC phenotypic switching and MØ polarization during atherogenesis. Detailed understanding of these transcriptional networks will enable us to develop novel diagnostic and therapeutic strategies to combat vascular proliferative diseases, including atherosclerosis.

## 1. Introduction

The chronic inflammatory disease atherosclerosis, which is characterized by atherosclerotic plaque formation in large and medium vessels, remains the major cause of morbidity and mortality in Western society. Atherosclerosis involves vascular inflammation and the accumulation of lipids, cholesterol, calcium, and cellular debris in the vessel wall. This leads to plaque formation, which causes vascular remodeling and obstructions, accompanied by abnormalities of blood flow and reduced oxygen supply to target organs [1,2,3,4,5]. Triggers that initiate atherosclerosis include oxidized low-density lipoproteins accumulating in the intima, as well as tissue damage that releases microbes and some endogenous molecules [6,7]. Scavenger receptors and Toll-like receptors (TLRs), present on endothelial cells (ECs), recognize and respond to these triggers by producing inflammatory cytokines, chemokines, and cell surface adhesion molecules. This process of EC dysfunction activates the successive recruitment and translocation of monocytes and lymphocytes from the circulation into the intima and mediates the early onset of atherosclerosis. Monocytes differentiate to lesional macrophages (MØ), which play prominent roles in chemokine/cytokine production, lesion matrix remodeling, cholesterol accumulation and foam cell formation, and clearance of dead cell debris [8,9]. Likewise, T helper 1 lymphocyte (Th1) subsets are important producers of pro-inflammatory cytokines, including TNFa and IL-12 and -18, and Type I interferon (IFN-I) and IFN-II (or IFNγ), all promoting atherogenesis [10]. Also, dendritic cells (DCs) have recently been identified as crucial components of atherosclerosis, as well as plaque destabilization [11]. Finally, altered contractility of Vascular Smooth Muscle Cells (VSMCs), mediated by lesional de-differentiation and proliferation and VSMC foam cell formation, contributes to vessel occlusion and neo-intima formation [12]. Collectively, these different cell types promote excessive inflammatory and immune responses and associated tissue damage and contribute to local inflammation, vascular damage, and remodeling of plaque lesions [1,2,3,4,5].

De-differentiation or phenotypic switching of VSMCs contributes to vascular remodeling and the pathogenesis of atherosclerosis [12]. Likewise, MØ plasticity and the presence of different phenotypes have a major effect on atherosclerotic plaque formation [8,9]. It has become clear that VSMCs and MØ plasticity are induced by numerous environmental cues and actively regulated by inflammation and inflammatory mediators. Accumulating evidence suggests that transcription factor networks involving Krüppel-like factor 4 (KLF4), Signal Transducer and Activator of Transcription (STAT), Interferon Regulatory Factor (IRF), and Nuclear factor-κB (NF-κB), in response to inflammatory factors, promote VSMC phenotypic switching and MØ polarization [4,13,14,15,16]. Detailed understanding of these transcriptional networks will enable us to develop novel diagnostic and therapeutic strategies to combat vascular proliferative diseases.

## 2. VSMC and MØ Plasticity in Atherosclerosis

### 2.1. VSMC Phenotypic Switching

**Figure 1:**
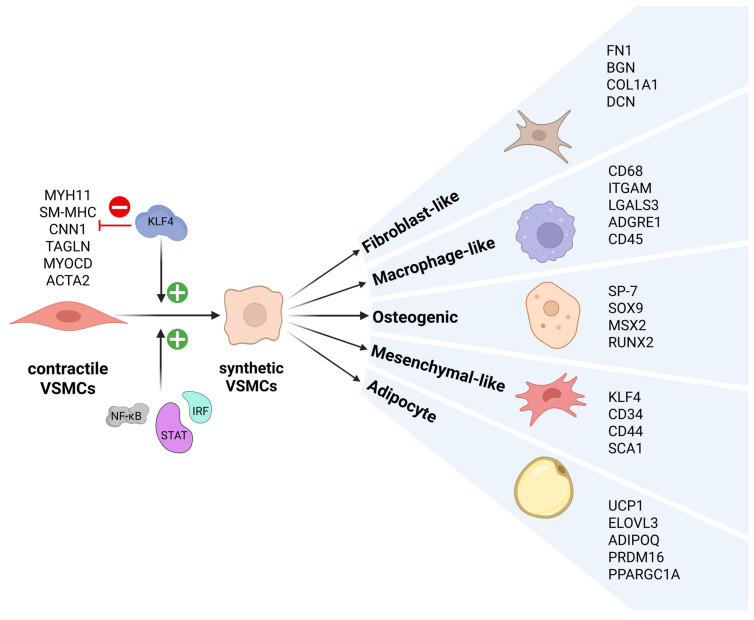
Phenotypic plasticity ofVSMCs. Contractile VSMCs, characterized by expression of *MYH11*, *SM*-*MHC*, *CNN1*, *TAGLN*, *MYOCD*, and *ACTA2*, can under specific conditions undergo de-differentiation into synthetic VSMCs with reduced expression of contractile markers. A key regulator of this initial switch is KLF4, a pluripotent transcription factor involved in repression of contractile gene expression (represented by a red “minus” icon). Additional regulators include STATs, IRFs, and NF-κB, which act as inflammatory response mediators and are also activated during this phenotypic transition, upregulating expression of inflammatory response genes (represented by a green “+” icon). Synthetic VSMCs, representing an intermediate de-differentiated state, demonstrate high plasticity and can further differentiate into multiple phenotypes, each exhibiting specific markers, indicated in brackets, including fibroblast-like (*FN1*, *BGN*, *DCN*, and *COL1A1*), macrophage-like (*CD45*, *CD68*, *LGALS3*, *ITGAM*, and *ADGRE1*), osteogenic (*SP*-*7*, *SOX9*, *MSX2*, and *RUNX2*), mesenchymal-like (*KLF4*, *CD34*, *CD44*, and *SCA1/LY6a*), and adipocyte-like (*UCP1*, *ELOVL3*, *ADI*-*POQ*, *PRDM16*, and *PPARGC1A*) VSMCs. This subsequent modulation is also regulated by KLF4, which, in contrast to its repressive role in the initial switch, activates expression of molecular markers of each specific subtype (represented by a green “+” icon).

Under normal physiological conditions, VSMCs regulate the vasomotor activity of blood vessels and adopt a contractile phenotype with low proliferative and synthetic activity. These VSMCs are also known as differentiated, quiescent, or contractile VSMCs. Apparently, VSMCs can be activated during the process of atherosclerosis and assume a proliferative, de-differentiated, or synthetic phenotype, exhibiting higher proliferative and synthetic activities (Figure 1). Contractile VSMC markers are exemplified by SM α-actin, myocardin (MYOCD), calponin (CNN1), transgelin (also known as SM22α), and MYH11 (also known as smooth muscle (SM) myosin heavy chain (SM-MHC)). The expression of contractile phenotype markers is diminished or lost, during transition of VSMC phenotypes, while synthetic markers are highly induced [13,17,18,19].

In addition to the two types of VSMCs mentioned above, recent single-cell RNA sequencing (scRNAseq) studies of human atherosclerotic plaques revealed such de-differentiated arterial VSMCs to be highly diverse (Figure 1). For example, Chen et al. described nine VSMC phenotypes that have been reported in atherosclerotic lesions and classified them into differentiated VSMCs, intermediately de-differentiated VSMCs, and de-differentiated VSMCs [19]. Accordingly, differentiated VSMCs may transition to intermediately de-differentiated VSMCs, constituting mesenchymal-like VSMCs, SEM-like cells, or Lgals3+ VSMCs, and then further to de-differentiated MØ-like VSMCs or osteoblast-like VSMCs. Or, differentiated VSMCs transform directly to de-differentiated VSMCs, including fibroblast-like VSMCs, myofibroblast-like VSMCs, MØ-like VSMCs, osteoblast-like VSMCs, or adipocyte-like VSMCs. Likewise, Zhang et al. and Yap et al. reported the classification of six VSMC phenotypes [13,17], with the mesenchymal-like phenotype categorized as the central de-differentiated VSMC type. Subsequent differentiation into MØ-like, fibroblast-like, adipocyte-like, and osteogenic-like VSMCs further modulates vascular disease. Mesenchymal phenotype marker genes include *CD34*, *CD44, KLF4*, and *SCA1/LY6a*, whereas *DCN*, *FN1*, *BGN*, and *COL1A1* are characteristic of the fibroblast phenotype. Typical marker genes for the MØ phenotype include *LGALS3*, *ITGAM*, *CD45*, *CD68*, and *ADGRE1*, and for the osteogenic phenotype *SOX9*, *MSX2*, *SP-7*, and *RUNX2*. Finally, the adipocyte phenotype is represented by the markers *ELOVL3*, *ADIPOQ*, *PRDM16*, *UCP1*, and *PPARGC1A*. A critical role for modulated VSMC phenotypes was also discovered by a meta-analysis study performed by Mosquera et al. They showed the presence of contractile VSMC, transitional VSMC, foam-like VSMC, and fibromyocytes, in CAD, myocardial infarction, and coronary calcification. Moreover, fibromyocyte/fibrochondrogenic VSMC markers were identified as proxies of atherosclerosis progression [20]. Although research has identified at least six main phenotypes (Figure 1), currently, no clear consensus exist about the number of VSMC phenotypes.

Interestingly, in advanced atherosclerotic lesions from both mice and human scRNAseq studies by Alencar et al. reported 14 different cell clusters and significant overlap between the VSMC clusters of the two species with respect to their functional categorization [21]. It has also become clear that phenotypic switching of VSMCs is induced by numerous environmental cues and actively regulated by inflammation and inflammatory mediators. Moreover, VSMCs can acquire an inflammatory phenotype within the lesion and also secrete inflammatory factors, contributing to local inflammation, vascular damage, and remodeling of plaque lesions [13,16,17,18,19].

### 2.2. MØ Polarization

**Figure 2:**
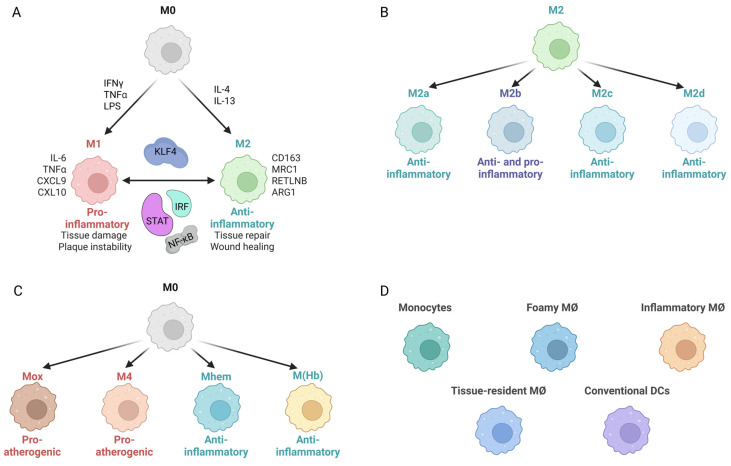
MØ polarization. (**A**) Classical MØ classification divides monocyte-derived macrophages into two main subtypes. Unactivated MØ, often referred to as M0, can respond to IFNγ, TNFα, and LPS and convert to M1 MØ, which are pro-inflammatory and contribute to tissue damage and plaque instability. In contrast, M0 MØ exposed to IL-3 and IL-4 polarize to an M2 phenotype, displaying anti-inflammatory properties that promote tissue repair and wound healing. M1 and M2 macrophages can switch in response to specific signals. Regulators involved in this process include KLF4, which can both activate and repress subtype-specific markers, along with STATs, IRFs, and NF-κB, which also have dual roles in controlling M1/M2 polarization. (**B**) Beyond the classical M1/M2 framework, macrophages can differentiate into specialized subtypes. M2 macrophages can be further categorized as M2a, M2b, M2c, and M2d, most of which are anti-inflammatory, except M2c, which displays both anti- and pro-inflammatory characteristics. (**C**) Additional specialized macrophages include Mox, M (Hb), Mhem, and M4, which are pro-, anti-, anti-, and pro-inflammatory, respectively. (**D**) Recent scRNA sequencing studies have revealed disease-relevant macrophage subtypes, including foamy macrophages, monocytes, inflammatory macrophages, tissue-resident macrophages, and conventional dendritic cells.

Many studies have shown that not only the number of infiltrated MØ but also MØ plasticity and the presence of different phenotypes have a major effect on atherosclerotic plaque formation [22,23,24,25]. Indeed, monocyte-derived MØ in the atherosclerotic plaque can adopt different subtypes with distinct functions (Figure 2A). A typical pro-inflammatory M1 phenotype is exhibited upon activation with TNFα, IFNγ, and TLR ligands, like lipopolysaccharide (LPS) (Figure 2A) [26,27]. This M1 MØ subtype is characterized by the secretion of pro-inflammatory cytokines and chemokines (CXCL9, CXCL10, CXCL11, IL-6, IL-12, IL-23, IFNγ, IL-1b, TNFa), reactive oxygen species (ROS) and nitric oxide (NO) [28,29], which induces tissue damage, and plaque instability impairs wound healing [30].

In contrast, Th2-associated cytokines, such as IL-4 and IL-13, induce MØ towards a more anti-inflammatory M2 phenotype. These cells are characterized by the presence of mannose receptor 1, resistin like-b, CD163, and high levels of arginase-1 [24] and promote tissue repair and wound healing, and to some extent inhibit disease progression (Figure 2A) [27,31,32]. Focusing on this MØ M1/M2 classification suggested a temporal and spatial component of MØ polarization in relation to atherosclerosis. Accordingly, the imbalance of the M1/M2 ratio in the atherosclerotic lesion is an important factor in the development of atherosclerosis.

Similar to VSMCs, during the last few decades MØ heterogeneity has gradually been recognized within the atherosclerotic plaques, whereas enhanced knowledge of MØ phenotypic diversity has been obtained through single-cell technologies [33,34,35,36]. For example, recent advances have suggested that M2 MØ can be further divided into M2a, M2b, M2c, and M2d subtypes, according to differing in vitro stimulation factors (Figure 2B) [8]. M2a promotes tissue repair and is referred to as wound healing MØ. The M2b subtype has both pro- and anti-inflammatory characteristics, while M2c and M2d both display strong anti-inflammatory activity. In addition, monocyte/MØ have been further divided into M4, Mox, M (Hb), and Mhem subtypes, with M4 and Mox being pro-atherogenic and the other two subtypes presenting a more anti-atherogenic phenotype (Figure 2C) [8,37]. Song at al. performed single-nucleus RNA sequencing of the carotid plaque tissue to further understand the mechanism of carotid atherosclerotic plaque formation. Accordingly, they identified 11 cell types, including five different MØ subpopulations, with the carotid plaques from patients with symptomatic atherosclerosis having reduced levels of M1-type MØ and increased levels of M2-, LAM-, and Mhem-type MØ compared to asymptomatic atherosclerosis [38]. Both Mosquera et al. [20] and Boroujeni et al. [20,39] performed an integrative meta-analysis using similar scRNAseq datasets from atherosclerotic lesions and non-lesion human coronary and carotid arteries. Thus, disease-relevant MØ subtypes were identified in human atherosclerosis, including conventional dendritic cells, foamy MØ, inflammatory MØ, tissue-resident MØ, and monocytes. Single-cell landscape studies of atherosclerotic plaques have recently been studied both in humans and mice [34,36]. This identified 13 distinct myeloid cell populations in mouse aortas with resident-like macrophages being found in both healthy and atherosclerotic aortas. On the contrary, several populations of macrophages, monocyte-derived dendritic cells, and monocytes were predominantly present in atherosclerotic aortas. A similar distribution was observed in human carotid plaques (Figure 2D). Although it has become clear that MØ can switch from one phenotype to another in the atherosclerotic plaque microenvironment [40], so far no consensus exists on the number of MØ phenotypes in atherosclerosis.

## 3. TLRs and IFNs in Atherosclerosis

The pro-inflammatory cytokine IFNγ and TLR4 stimuli are among key factors contributing to the onset and progression of atherosclerosis. TLRs are developed to detect molecules associated with microbial infection or tissue damage and initiate inflammatory signaling. This results in the rapid expression of multiple inflammatory mediators, like inflammatory cytokines, chemokines, and endothelial adhesion molecules, in most cell types [4,41]. Dysregulated TLR signaling contributes significantly to the development of atherosclerosis [42,43,44], specifically with the expression of TLR4 detected in ECs and MØ, as well as VSMCs of both human and mouse atherosclerotic lesions [45]. Likewise, increased TLR4 expression on circulating monocytes was observed in patients with coronary arteriosclerotic lesions compared with control patients [46]. In a Apolipoprotein E Knockout (ApoEKO) mouse model, TLR4 deficiency reduced atherosclerosis upon a high fat diet (HFD), confirming a crucial role of TLR-dependent pathways in disease development [47,48]. A combined role of TLR4-dependent signaling in vascular cell activation and dysregulation of MØ cholesterol metabolism was shown as a prerequisite for the formation of foam cells and lesion progression in vivo [49,50]. Enhanced TLR4 signaling was also shown to promote VSMCs to switch from a contractile phenotype to a pro-inflammatory phenotype, resulting in the synthesis of pro-inflammatory mediators [16,41,51,52,53,54]. In addition, TLR4 signaling promotes M1 polarization and TLR4 activation promotes pro-inflammatory M1 MØ polarization, leading to cytokine production and immune responses. Conversely, TLR4 deficiency can lead to alternative, anti-inflammatory M2 polarization, shifting the macrophage metabolic profile [16,41,42,43,44,50].

As a member of the IFN family, IFNγ is a vital modulator of both innate and adaptive immunity by activating MØ, natural killer cells, B cells, and ECs and VSMCs [55,56]. IFNγ, primarily produced by T cells, natural killer cells, and MØ, has emerged as an important factor in atherogenesis [57,58,59,60]. As such, multiple cells of the innate and adaptive immune response are activated by IFNγ in the local plaque environment, triggering the production of multiple cytokines, growth factors, interleukins, chemokines and adhesion molecules. This enables monocyte recruitment to the endothelial wall, intimal translocation, and differentiation into the MØ M1 pro-inflammatory phenotype. At the same time, expression of scavenger receptors is stimulated by IFNγ in MØ, while reverse cholesterol transport proteins are suppressed. This promotes accumulation of modified low-density lipoprotein (mLDL) and suppression of cholesterol efflux to HDL and leads to foam cell formation. Successive intimal lipid overload facilitates plaque core necrosis and ECM degradation [61]. Finally, the transition of VSMCs from a contractile to a proliferative phenotype is another important process of atherosclerotic plaque progression induced by IFNγ [16,62]. The atheroma-promoting properties of IFNγ are also supported by a multitude of murine studies. After 8 weeks on a HFD, LDL receptor (LDLr) KO mice deficient in IFNγ exhibited a reduction in atherosclerosis in the aortic arch and descending aorta as compared to LDLrKO mice [63]. The same result could be observed in mice with double KO of ApoE and the IFNγ receptor after 3 months on a HFD [64]. Although IFNγ-KO mice were protected from developing coronary arteriosclerosis, both wild-type (WT) and IFNγ-KO mice given heart transplants developed myocardial rejection [65]. A similar protection of atherosclerosis could also be detected in WT mice treated with anti-IFNγ antibodies upon heart transplantation. Increasing atherosclerotic lesion size and MØ and T cell recruitment upon exogenous IFNγ administration have also been observed in other murine studies [66].

## 4. STATs, IRFs, and NF-κB in IFNγ and TLR4 Signaling

**Figure 3:**
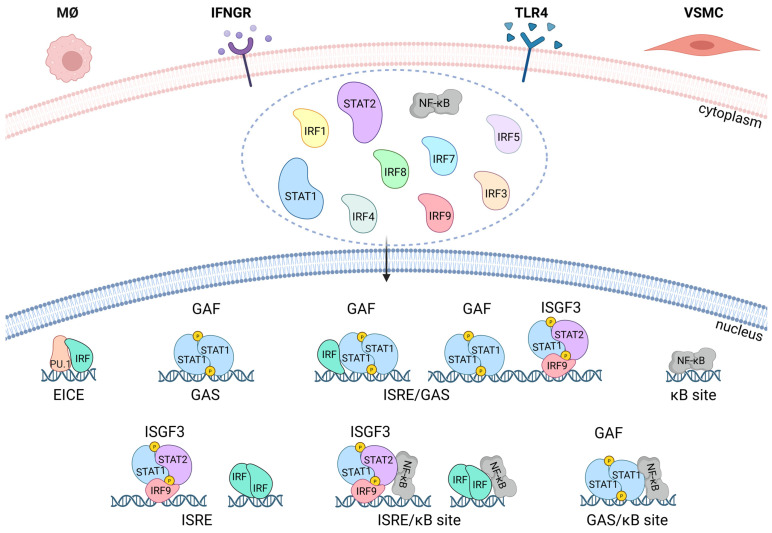
Transcriptional responses and signal integration of IFNγ and TLR4 pathways in VSMCs and MØ. Transcription factors, including STAT1, STAT2, multiple IRFs, NF-κB, and PU.1, are regulators involved in driving the inflammatory response in response to external stimuli, recognized by IFNGR and TLR4, and are shared components of the signaling networks in VSMCs and MØ. Ligand binding to these receptors results in the activation and translocation of the transcription factors from the cytoplasm to the nucleus, where they interact in a coordinated manner. The classical IFNγ-dependent signaling in response to IFNGR activation occurs through STAT1 phosphorylation and formation of a homodimer known as gamma-activated factor (GAF), which regulates the expression of gamma-activated sequence (GAS)-containing genes. Alternatively, in response to IFN-γ, a complex called interferon-stimulated gene factor 3 (ISGF3), composed of IRF9 and phosphorylated STAT1 and STAT2, as well as multiple IRFs, have been shown to regulate transcription of genes containing interferon-stimulated response elements (ISRE). LPS ligation of TLR4 activates NF-κB and IRFs, leading to a rapid inflammatory response and the induction of genes, including IFN-I and TNFα. They initiate a secondary response wave that establishes a positive feedback loop, resulting in further activation of STATs, IRFs, and sustained NF-κB activity. NF-κB regulates the expression of genes with κB-binding sites. Additionally, these factors can also cooperate to activate a combination of elements, such as ISRE/GAS, ISRE/NF-κB, or GAS/κB sites. Signal integration of TLR4 and IFN-γ pathways synergistically amplifies transcriptional programs by combining TLR4-driven NF-κB/IRF activation with IFN-γ–induced STAT1 signaling, resulting in an enhanced inflammatory response. Furthermore, in MØ a distinct mechanism is involved, in which PU.1, a MØ-specific lineage-determining transcription factor, in cooperation with IRFs, activates a distinct set of Ets–IRF composite elements (EICEs), present in immune regulatory genes.

STAT, IRF, and NF-κB transcription factor families facilitate action of interferons, cytokines, growth factors, and pathogens and play a crucial role in innate and adaptive immunity and during inflammation (Figure 3) [67]. IFNγ treatment of multiple cell types, including VSMCs and MØ, results in the formation of STAT1 homodimers, known as γ-activated factor (GAF), and direct activation of transcription of IFNγ-activated sequence (GAS; consensus TTTCNNNGAAA)-containing target genes [55,68]. Additionally, IFNγ is able to activate genes through a second STAT-based signaling cascade, entailing the formation of STAT1-STAT2 heterodimers in association with IRF9 [known as interferon-stimulated gene factor 3 (ISGF3)], which then promote the expression of a distinct set of ISRE (IFN-stimulated response element: consensus AGTTTCN_2_TTTCN)-containing genes. Although this second pathway is primarily activated by IFN-I [55,68], support for a direct role of ISGF3 in IFNγ signaling was reported in mouse primary embryonic fibroblasts (MEF) [69]. Additionally, IRF9KO mice were impaired in both the IFN-I and IFNγ transcriptional response of ISRE-containing genes [70]. Similarly, in MEFs, the antiviral potency of IFNγ depended on STAT2 phosphorylation [71]. Recently, evidence was provided for a more genome-wide role of ISGF3 in the IFN-I and IFNγ-activated responses of mouse MØ and fibroblasts by Platanitis et al. [72]. Our group observed combined recruitment of pSTAT1, pSTAT2, and IRF9 to ISRE-containing genes in mouse VSMCs treated with IFN-I and IFNγ [16]. Likewise, IRF1, IRF5, IRF7, and IRF8 have been shown to regulate transcription of ISRE-containing genes in response to IFNγ in both VSMCs and MØ [16,41,73]. Together, this points to the existence of STAT- and IRF-dependent mechanisms by which IFNγ can elicit activities in vascular and immune cells (Figure 3).

TLR ligation in VSMCs and MØ and many other cell types results in the rapid activation of members of the NF-κB and IRF families, including p50 and p65 and IRF1, IRF3, IRF4, IRF5, IRF7, and IRF8 (Figure 3) [41,67]. These factors work in a combinatorial manner to rapidly induce the expression of hundreds of genes, with NF-κB binding to variations in the consensus DNA sequence of 5′-GGGRNYY YCC-3′, known as κB sites [74]. This results in amplification of the initial inflammatory response, mediates antimicrobial activities, and initiates the development of adaptive immunity [75,76]. It also results in the rapid induction of genes, including IFN-I and TNFα, which initiate a secondary response wave that establishes a positive feedback loop, resulting in further activation of STAT1 and STAT2, multiple IRFs, and sustained NF-κB activity.

VSMC and MØ, in general, perform different and rather unique functions. However, during vascular inflammation and onset and progression of atherosclerotic plaques, these two cell types also exhibit common functions, like lipid uptake and excessive release of pro-inflammatory stimuli. Under these conditions, regulation of expression of many IFNγ-activated pro-inflammatory genes relies on Signal Integration (SI) with TLR4 (Figure 3) [15,16,41]. Accordingly, IFNγ and TLR4 participate in signaling cross-talk through combinatorial transcription factor interactions on GAS, ISRE, ISRE/NF-κB, GAS/NF-κB, or ISRE/GAS binding sites. As such, robust expression of multiple chemokines, adhesion molecules, and antiviral and antimicrobial proteins in vascular and immune cells is coordinated by combined action of STATs, NF-κB, and different IRFs (Figure 3) [4,16,41,77,78,79,80,81,82,83]. Likewise, IFNγ-induced co-binding of IRF1, IRF8, STAT1, and the lineage-determining transcription factor PU.1 to ISRE-containing genes in MØ directs the expression of a set of genes, the IRF8/IRF1 regulome, that play critical roles in host inflammatory and antimicrobial defenses (Figure 3) [73].

## 5. STATs, IRFs and NF-κB in Inflammation and Atherosclerosis

Multiple studies also provide evidence for a role of STATs, IRFs, and NF-κB in atherosclerosis. In an intraperitoneal inflammation and an atherosclerosis-susceptible bone marrow transplantation mouse model, Agrawal et al. identified a regulatory role of STAT1 in foam cell formation and atherosclerotic lesion development [84]. STAT1 was shown to be critical for MØ apoptosis, responsible for the necrotic core formation in atherosclerotic plaques [85]. Similarly, STAT1 activity was associated with VSMC de-differentiation and decreased expression of contractile genes [86], as well as proliferation of VSMCs and neointimal hyperplasia [87]. Furthermore, in VSMCs and ECs of human atherosclerotic plaques, elevated expression of the chemokines CXCL9 and CXCL10 correlated with phosphorylated STAT1 [81]. After performing data mining of human plaque transcriptomes, we recognized increased expression of a selection of proatherogenic STAT1-target genes, including *CRCL2*, *Cd74*, *CXCL9*, *CXCL10*, *CCL5*, *CCL8*, *IRF1*, and *IRF8* in human plaques from carotid and coronary arteries [15]. We also recently identified ALEKSIN as a novel multi-IRF inhibitor, which, similarly to the known multi-STAT inhibitor STATTIC, exhibited genome-wide inhibition of STAT-, IRF-, and NF-κB-target genes [88]. More importantly, a signature of 46 pro-atherogenic genes, commonly inhibited by ALEKSIN and STATTIC, could be predominantly linked to MØ subtypes present in aortic plaques in HFD-fed LDLR-KO mice [88]. According to a multi-omics workflow, we integrated RNA-seq, ChIP-seq, and ATACseq data from IFNγ-treated primary mouse MØ and identified a subset of STAT1- and PU.1-target genes. Combined with sc- and bulk RNAseq data from human and mouse atherosclerotic lesions, we observed dynamic transcriptional changes in these genes in MØ and VSMC subtypes [39]. We also concluded that VSMC- and MØ- expressing STAT1-target genes reflected unique functional subsets present in human atherosclerotic and non-atherosclerotic arteries. Based on this, a novel STAT1-dependent gene signature to monitor “MØ-dependent” plaque progression during human atherosclerotic disease could be selected [39].

Impaired activity of IRFs is also implicated in atherosclerosis [89]. For example, silencing IRF1 alleviated atherosclerosis in ApoEKO mice by regulating lipid metabolism and foam cell formation [90]. Particularly, a role for IRF1 was shown in the regulation of gene expression implicated in foam cell formation in VSMCs and MØ. IRF1 also contributed to the pathological phenotype of VSMCs during atherogenesis by increasing CCL19 transcription [91]. Moreover, ablation of IRF3 protected against atherosclerosis in ApoEKO mice [92]. Similarly, a role of IRF5 was suggested in vulnerable and symptomatic human carotid plaques, and in hyperlipidemic ApoEKO mice experiencing plaque rupture. By impairing efferocytosis in atherosclerotic plaques, IRF5 was also shown to control necrotic core formation. However, MØ-specific IRF5 deficiency was shown to stabilize atherosclerotic plaques in ApoEKO mice [93,94,95]. On the other hand, IRF4 protects arteries against neointima formation upon vascular injury [96]. The same is true for IRF7, which has shown to protect against VSMC proliferation and neointima formation [97]. *IRF8* gene single nucleotide polymorphisms were associated with carotid plaques and increased intima-media thickness [98,99]. Döring et al. revealed that IRF8KO bone marrow transplantation into ApoEKO mice exacerbated atherosclerotic lesion formation [100]. Other studies reported that IRF8 was crucial in modulating VSMC phenotype switching and neointima formation in response to vascular injury via direct interaction with the SRF/MYOCD complex [101]. Likewise, a critical role for IRF9 was shown in mediating neointima formation following vascular injury [102]. IRFs are also crucial in directing MØ polarization towards M1 or M2 phenotypes. IRF1, IRF5, and IRF8 typically promote the M1 phenotype by enhancing pro-inflammatory gene expression, while IRF3 and IRF4 are associated with the M2 phenotype, often by opposing M1-driving signals or promoting M2-related genes [103,104].

NF-κB also acts as a critical transcription factor in atherosclerosis, promoting inflammatory processes at every stage of plaque development by regulating the expression of genes for cytokines, chemokines, adhesion molecules, and other factors that recruit immune cells and contribute to plaque formation and progression [105,106,107]. Location of p65 and p50 was shown in vessel nuclei in response to arterial injury as well as in human atherosclerotic lesions, including ECs, MØ, and VSMCs. Moreover, NF-κB inhibition diminished vascular injury-mediated neointima formation [108]. Interestingly, endothelium-specific ablation of NF-κB in ApoEKO mice on a HFD resulted in severely diminished atherosclerotic plaque formation [109]. Meanwhile, hematopoietic cell-specific knockout of the *p50* gene in LDLRKO mice resulted in smaller atherosclerotic lesions [110]. Oppositely, myeloid-specific deletion of IκB in these mice displayed larger and more advanced atherosclerotic lesions [111]. Finally, inhibition of NF-κB in a VSMC-selective manner reduced vascular injury-dependent VSMC phenotypic switching and neointima formation [112]. With regard to MØ polarization, NF-κB acts as a master regulator, with its activation promoting the pro-inflammatory M1 phenotype and the induction of a large number of inflammatory genes [105,106,107]. Together, these studies provide compelling evidence for a crucial role of NF-κB activation within ECs, MØ, and VSMCs during vascular proliferative diseases. Consequently, this marks NF-κB as a potential therapeutic target for vascular diseases including atherosclerosis.

## 6. KLF4 in VSMC Phenotypic Switching: Promotion of a Pro-Atherogenic Phenotype, Inhibition of Neointima Formation

**Figure 4:**
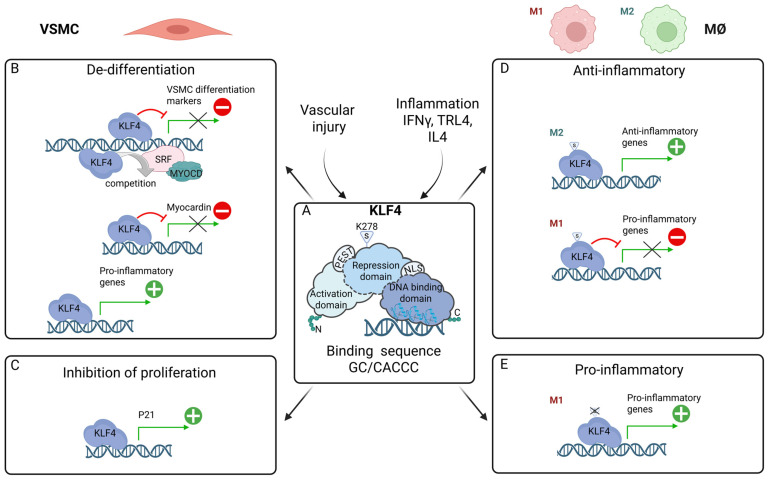
KLF4 in VSMC phenotypic switching and MØ polarization. (**A**) KLF4 is a pluripotent transcription factor. Its N-terminus contains a transcriptional activation domain (TAD) and a repression domain, which together control KLF4 activity. The C-terminus contains a DNA-binding domain (DBD), composed of three zinc fingers, which recognizes the GC/CACCC sequence. In addition, KLF4 has a PEST sequence and a nuclear localization signal, and its transcriptional activity is regulated by SUMOylation at lysine 278 (represented by K278: S). In response to inflammatory factors and vascular injury, KLF4 is involved in the control of VSMC de-differentiation and MØ polarization. (**B**) KLF4 contribution to VSMC de-differentiation occurs in two distinct ways. In one direction, it acts as a repressor (represented by a red “minus” icon) of VSMC differentiation markers, either by directly binding to the promoters/enhancers of these genes, or by competing with SRF, a potent activator of these VSMC genes, or through inactivation of MYOCD, an SRF coactivator. In the second direction, KLF4 can function as an activator (represented by a green “+” icon) of pro-inflammatory genes, which contributes to further VSMC de-differentiation. (**C**): KLF4 is also involved in mechanisms of cell-cycle arrest by directly activating the expression of *p21*, leading to reduced proliferation. (**D**) In response to M2 stimuli (IL-4 and IL-13), KLF4 drives the anti-inflammatory response. When SUMOylated, in M2 MØ, KLF4 activates the expression of anti-inflammatory genes (represented by a green “+” icon); in M1 MØ, it represses pro-inflammatory genes (represented by a red “minus” icon). Both mechanisms contribute to polarization toward the M2 phenotype. (**E**): Under de-SUMOylation, KLF4’s control over pro-inflammatory genes shifts from repression to activation, promoting M1 polarization (represented by a green “+” icon).

KLF4 is a member of a zinc finger transcription factor family with known functions in cellular development, apoptosis, differentiation, and proliferation,. KLF4 is also critical for the generation of induced pluripotent stem cells as one of the four Yamanaka reprogramming factors [113]. KLF4 is built of three distinct domains: (1) a carboxyl terminal DNA-binding domain (DBD), harboring three zinc finger motifs; (2) an amino terminal Transcriptional activation domain (TAD); and (3) a centrally located repression domain [114,115]. Together they control the multi-regulatory characteristics of KLF4, as a transcriptional repressor or activator, by modulating DNA binding efficiency and interaction with other proteins (Figure 4A).

Two nuclear localization signals (NLSs) have also been identified in mouse KLF4. Potential PEST sequences can be recognized as well, facilitating degradation by the ubiquitin–proteasome pathway. KLF4 is a SUMOylated protein and its SUMOylation plays a major role in regulating the transcriptional activity. Recent studies have identified KLF4 as a 5mC reader in mouse embryonic stem (ES) cells and suggested that KLF4 binds to the consensus DNA sequence GC/CACCC [116,117] via its zinc-finger motifs, interacting with co-activators and co-repressors, and influencing the chromatin structure to either activate or suppress the transcription of specific genes (Figure 4A).

As mentioned above, the expression of contractile phenotype markers like SM a-actin, SM22 a, and SM-MHC is diminished or lost during VSMC phenotypic switching [13,118,119]. Promoters/enhancers of these VSMC differentiation markers share the presence of multiple CC(A/T-rich)6GG (CArG) elements and a transforming growth factor (TGF)-b control element. Through specific recruitment of SRF to CArG elements, together with its coactivator MYOCD or its corepressor phosphorylated Elk-1, expression of VSMC differentiation marker genes is regulated. Upregulation of the *MYOCD* and *SRF* genes is facilitated by binding of the DNA-modifying enzyme Tet methylcytosine doxygenase 2 (TET2, also known as ten-eleven translocation-2) to their CArG-rich regions, and results in subsequent induction of downstream VSMC contractile genes. TET2 reduction in de-differentiated VSMCs leads to a concomitant transcriptional upregulation of *KLF4* [13,118,119].

In the context of VSMC phenotypic switching, in response to inflammatory factors like cytokines, growth factors, and oxidized phospholipids (ROS damage), Klf4 acts as a potent repression of VSMC differentiation markers (Figure 1) through various mechanisms (Figure 4B; represented by a red “minus” icon): (1) Klf4 directly binds to the TGF-β control element; (2) KLF4 interacts with SRF recruited to CArG elements; and (3) KLF4 represses expression of *MYOCD* [120,121]. Interestingly, transition from contractile to mesenchymal-like VSMCs depends on KLF4, by downregulating contractile markers and at the same time initiating the expression of mesenchymal markers such as stem cell antigen-1 (Figure 1; represented by a green “+” icon) [13]. Likewise, a potentiating role of KLF4 has been proposed in other VSMC phenotypes, including Fibroblast-, MØ-, Osteogenic-, and adipocyte-like (Figure 1), via a variety of regulatory mechanisms [13].

As an inhibitor of DNA synthesis and cellular growth in a variety of cells, KLF4 has also been reported as a central transcriptional regulator of cellular proliferation [114,115]. For example, in response to DNA damage, KLF4 is upregulated and in combination with P53 facilitates cell-cycle arrest at the G1/S checkpoint by binding to the promoter of the cyclin-dependent kinase inhibitor *P21* and promoting its transcriptional activation (Figure 4C; represented by a green “+” icon) [122]. Likewise, cell growth inhibition of cultured VSMCs was mediated by KLF4-induced P21 [123].

Additional studies provide evidence for this dual regulatory role of KLF4 in vascular injury in vivo. For example, Liu et al. observed transiently induced expression of *KLF4* mRNA in rat carotid arteries after balloon injury [121]. Studies by Yoshida et al. were in line with detecting rapid induction of KLF4 expression in VSMCs of WT mice as compared to *KLF4* VSMC-specific conditional KO mice, after vascular injury. Under the same conditions, a transient delay of VSMC differentiation gene repression occurred in Klf4KO mice. These mice also exhibited enhanced neointimal formation caused by increased cellular proliferation, whereas cultured VSMCs overexpressing KLF4 showed reduced cellular proliferation depending on P21 [124]. Similarly, Salmon et al. showed that after vascular injury, KLF4 binding to the G/C Repressor element was responsible for injury-induced repression of the *SM22a* gene [125].

Moreover, in the HFD ApoEKO mouse atherosclerosis model, VSMC-specific conditional KO of KLF4 resulted in reduced lesion size, increased fibrous cap thickness, and major reductions in the fraction of VSMC-derived MØ- and MSC-like cells. In contrast, an increase in the fraction of ACTA2+ cells was observed within the fibrous cap. Moreover, cultured VSMCs loaded with cholesterol stimulated KLF4-dependent activation of MØ and MSC markers, accompanied by expression of pro-inflammatory cytokines and increased phagocytic characteristics. Finally, more than 800 putative KLF4-regulated genes were identified in VSMCs using in vivo KLF4 ChIP-seq analyses, including many associated with pro-inflammatory processes (Figure 4B; represented by a green “+” icon). Collectively, this provides convincing evidence for a pro-atherogenic function of KLF4 within VSMCs [126]. Follow-up studies by Alencar et al. [21] provided additional proof for a possible mechanism, in which KLF4 expression in VSMCs induces a phenotype shift towards a Lgals3+ osteogenic phenotype that is likely to be harmful for late-stage atherosclerotic plaque progression (Figure 1; represented by a green “+” icon) [127].

## 7. KLF4 in MØ Polarization: Multi-Functional Regulator of Pro- and Anti-Inflammatory Phenotypes

Multiple in vitro and in vivo findings support an important role for KLF4 as a regulator of MØ polarization [14,128,129,130]. Several studies in murine MØ suggest that KLF4 predominantly promotes the anti-inflammatory M2 phenotype (Figure 4D; represented by a green “+” icon). In response to M2 stimuli (IL-4, IL-13), KLF4 expression is robustly induced and expression remains high in the anti-inflammatory M2 MØ, promoting the expression of M2 target genes, including *Arg1*, *Refnla*, and *Pparg* [131]. This is accompanied by KLF4 SUMOylation at position lysine 278, which is conserved in different species (Figure 4D) [132]. Conversely, KLF4 expression is suppressed by M1 polarization stimuli (LPS, IFNγ) and KLF4 levels in pro-inflammatory M1 MØ are low. Under these conditions, KLF4 inhibited expression of pro-inflammatory genes, including *MCP-1*, *RANTES*, iNOS, *VCAM1*, and *IL1β*, and *TNFα* (Figure 4D; represented by a red “minus” icon) [131]. On the other hand, de-SUMOylation of KLF4, mediated by the small ubiquitin-like modifier (SUMO) specific peptidase (SENP)1, was vital in M1 MØ polarization [133]. In addition, myeloid-specific KLF4KO mice were predisposed to developing diet-induced glucose intolerance, insulin resistance, and obesity, and displayed delayed wound healing [134]. Also, KLF4KO MØ displayed enhanced pro-inflammatory activation and foam cell formation in response to oxidized lipids. In vivo, myeloid KLF4KO mice on a ApoEKO background kept on a HFD developed significantly more vascular inflammation and atherosclerotic lesion formation [134]. The anti-inflammatory effects of KLF4 were confirmed by two studies of Liu et al. in RAW264.7 MØ. On the one hand, LPS-induced KLF4 expression was shown to inhibit the expression of *IL-1β* in RAW264.7 macrophages [135]. Under similar conditions, KLF4 promoted the transcription of *IL-10* by binding to the IL-10 promoter [136].

Previously, the SENP1-KLF4 axis was identified to play a crucial role in regulating LPS-induced MØ M1 polarization (Figure 4E). Interestingly, KLF4 SUMOylation KO MØ demonstrated increased pro-inflammatory gene expression and enhanced bactericidal effects in response to LPS [133]. In line with this, Feinberg et al. revealed in MØ that KLF4 was markedly induced in response to IFNγ, LPS, or TNFa. Moreover, overexpression of KLF4 in J774a MØ resulted in upregulation of the MØ M1 activation marker inducible nitric-oxide synthase (iNOS) (Figure 4E; represented by a green “+” icon). Conversely, KLF4 knockdown markedly attenuated the ability of IFNγ, LPS, or IFNγ together with LPS to induce transcriptional activity of the *iNOS* promoter, which was mediated by two KLF DNA-binding sites [137]. It was also found that KLF4 overexpression in RAW 264.7 cells promoted M1 polarization. In vivo, KLF4 overexpression further intensified chondrocyte injury, augmented apoptosis, and stimulated inflammatory joint injury [138]. Rosenzweig at al. found that KLF4 acts in a dual function manner in the production of IL-6 by dendritic cells in response to LPS. It binds to and activates the *IL-6* promoter and has a role in the chromatin remodeling of the *IL-6* promoter [139]. Similarly, KLF4 was shown to be a regulator of pro-inflammatory signaling in fibroblast-like Synoviocytes through increased IL-6 expression [140].

Together, these studies point to a crucial role of KLF4 in MØ differentiation upon inflammatory stimuli, demonstrating pro- as well as anti-inflammatory properties (Figure 4D,E). Nevertheless, whether KLF4 acts predominantly in a pro- or anti-inflammatory manner in MØ is currently not clear [14]. However, KLF4 polarizing activity in MØ to the anti-inflammatory M2 subtype contradicts a shift towards a pro-atherogenic migrating and proliferating phenotype in VSMCs. Both polarizing events depend on the KLF4-galectin-3 axis, implying that VSMC-specific targeting of KLF4/galectin-3 function could be beneficial as a therapeutic strategy in the treatment of atherosclerosis [141].

## 8. Interplay Between KLF4, STATs, IRFs, and NF-κB in Inflammation and Atherosclerosis

**Figure 5:**
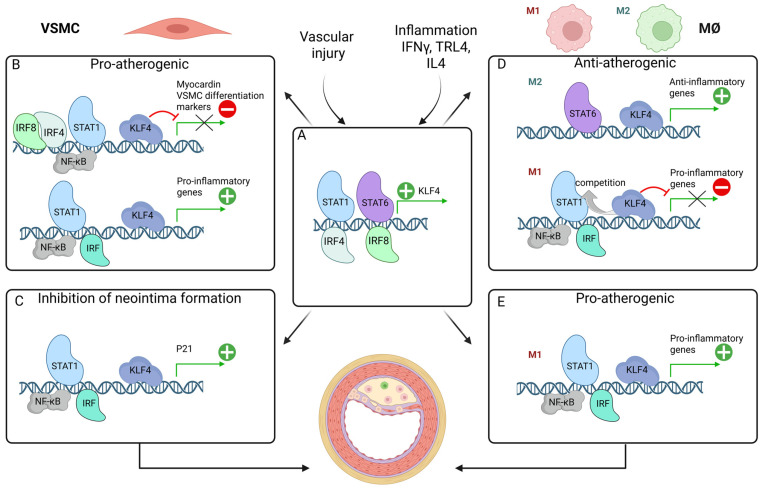
KLF4, STATs, IRFs, and NF-κB in inflammatory modulation. (**A**) In response to vascular injury and inflammatory stimuli, STAT1, STAT6, IRF8, and IRF4 have been shown to bind the KLF4 promoter, activating its expression (represented by a green “+” icon). (**B**) Multiple factors regulate the pro-atherogenic phenotypic switching of VSMCs. One mechanism involves STAT1, whose binding sites are present in MYOCD and VSMC differentiation marker genes. STAT1 possibly collaborates with KLF4 to repress gene expression (represented by a red “minus” icon), promoting VSMC de-differentiation. Similarly, KLF4 cooperates with p65 to bind MYOCD, and IRF8 and IRF4 further enhance KLF4’s repressive effect as co-binders. In the opposite direction, KLF4, STAT1, various IRFs, and NF-κB cooperate to activate expression of pro-inflammatory genes (represented by a green “+” icon). Importantly, IRFs and STATs contribute to VSMC de-differentiation not only by co-binding with KLF4 but also by directly activating its expression, amplifying the regulatory loop (**A**): represented by a green “+” icon). (**C**) A cooperative mechanism by which STAT1, IRFs, NF-κB, and KLF4 inhibit neointima formation involves mutual targeting of *p21*, leading to its transcriptional activation, resulting in suppression of VSMC proliferation (represented by a green “+” icon). (**D**) The cooperative action of STATs, IRFs, NF-κB, and KLF4 is also observed in the regulation of anti-atherogenic modulation in MØ, where it presents dual activities. In M2 MØ, this action is in a positive regulation of anti-inflammatory genes, with STAT6 partnering with KLF4 to stimulate their expression (represented by a green “+” icon). Conversely, in M1 MØ, KLF4 can inhibit the pro-inflammatory response through competitive binding, attenuating the activating roles of IRFs, NF-κB, and possibly STATs at pro-inflammatory gene promoters (represented by a red “minus” icon). (**E**) In the absence of KLF4 SUMOylation, its mode of action shifts from inhibitory to activating (represented by a green “+” icon). Under these conditions, KLF4 has been shown to activate pro-inflammatory gene expression in M1 MØ, cooperating with STATs, NF-κB, and IRFs.

As outlined above, KLF4 acts as a multi-functional transcriptional regulator of VSMC phenotypic switching and MØ polarization, inflammation, and progression of atherosclerosis. From recent studies, it also becomes clear that KLF4 collaborates with STATs, NF-κB, and IRFs in different, cell-type specific transcriptional mechanisms, contributing to plaque progression or regression (Figure 5).

### 8.1. Potentiating VSMC Phenotypic Switching: Pro-Atherogenic

Among the KLF4-target genes are VSMC-differentiation genes, including *SMα-actin*, *SM22α*, and *SM-MHC*, where KLF4 acts as a potent transcriptional repressor [13,118,119]. STAT1 and STAT3 have been shown to play opposing roles in modulating VSMC phenotype. Increased levels of STAT1 promotes VSMC de-differentiation, whereas high levels of STAT3 drives VSMC into a more mature phenotype [86]. Analyses of the promoter/enhancer region show that STAT binding elements are present in many VSMC contractile genes and that ChIP assays confirm binding of STAT1 and STAT3 to the calponin promoter. Under these conditions, STAT3 was shown to interact with MYOCD [142]. It can therefore be speculated that STAT1 cooperates with KLF4 to repress VSMC differentiation marker genes or that regulation is achieved by interfering with MYOCD or SRF binding (Figure 5B; represented by a red “minus” icon). Alternatively, it is possible that the mechanism by which STAT1 downregulates VSMC contractile gene expression is indirectly, through regulating the expression of *KLF4* (Figure 5A; represented by a green “+” icon). Indeed, in colon cancer cells, *KLF4* was recently identified as one of the downstream targets of IFNγ and STAT1, mediated through interaction of STAT1 with a GAS element present in the *KLF4* promoter (Figure 5A) [143].

Using the mouse left carotid artery wire injury model, it was observed that the expression of IRF8 was greatly enhanced in VSMCs upon injury. Compared with WT controls, IRF8KO mice exhibited reduced neointimal lesions and maintained VSMC marker gene expression. In contrast, a synthetic phenotype and enhanced neointima formation was observed in SM22a-driven VSMC-specific IRF8 transgenic (TG) mice. Specifically, IRF8 regulated SRF transactivation and inhibited VSMC marker gene expression, via direct interaction with MYOCD. In this way, IRF8 could potentiate the repressor effect of KLF4 on *MYOCD* transcription (Figure 5B; represented by a red “minus” icon) [101].

Results of previous studies have shown that inhibition of the NF-κB pathway reduces neointima formation following vascular injury. As such, in SM22a-driven VSMC-selective truncated IκB-expressing mice, in which NF-κB was inhibited selectively in VSMCs, neointima formation was markedly reduced after injury [112]. Reduced downregulation of VSMC differentiation marker and *MYOCD* expression was also observed. In cultured VSMCs, NF-κB activation by IL-1β resulted in decreased expression of VSMC differentiation markers as well as *MYOCD*, accompanied by KLF4 and p65 promoter binding. This *MYOCD* promoter binding was also detected in the carotid arteries following vascular injury. These results predict that KLF4 cooperates with p65 and contributes to VSMC phenotypic switching towards an inflammatory state (Figure 5B; represented by a red “minus” icon) [112]. A similar cooperative mechanism between KLF4 and p65 was shown in a mouse model of chronic kidney disease (CKD) with arterial medial calcification, driving phenotypic switching of VSMCs into osteogenic cells [144].

As mentioned earlier, VSMC-specific conditional KO of *KLF4* resulted in reduced lesion size, increased fibrous cap thickness, and major reductions in the fraction of VSMC-derived MØ- and MSC-like cells. In this context, more than 800 putative KLF4-target genes were identified in VSMCs, including many associated with pro-inflammatory processes [126]. These genes also contain potential STAT, IRF, and NF-κB targets, and predict the existence of a novel transcriptional regulatory circuit, depending on the cooperation of KLF4 with STATs, IRFs, and NF-κB in promoting inflammatory gene expression during VSMC phenotypic switching and vascular inflammation (Figure 5B; represented by a green “+” icon).

### 8.2. Inhibition of Neointima Formation

In the context of VSMC proliferation, KLF4 was shown to promote a cell-cycle arrest at the G1/S checkpoint through transcriptional activation of the cyclin-dependent kinase inhibitor P21 [124]. Interestingly, expression of IRF4 could be detected in VSMCs of mouse and human restenotic arteries, while complete IRF4 KO resulted in neointima formation in rats as well as mice [96]. Consequently, a thicker neointima developed in VSMC-specific IRF4-KO mice after injury as compared to control mice, complemented by increased VSMC proliferation and migration. With VSMC-specific *IRF4*-TG mice exhibiting the reverse phenotype, this demonstrates a protective role of IRF4 against neointima formation. Mechanistically, IRF4 promoted *KLF4* expression through direct promoter binding, indicating that these protective effects of IRF4 are KLF4-dependent (Figure 5A; represented by a green “+” icon) [96]. This leads to increased P21 expression and inhibition of VSMC proliferation. In addition, IRF4 promoted VSMC de-differentiation. With *P21* being a known target gene of STAT1, NF-κB, and multiple IRFs [145,146,147], and essential for cell growth suppression in response to IFNγ and other inflammatory signals, a cooperative mechanism between KLF4 and STAT1, IRF, and NF-κB can be predicted that affects VSMC proliferation and neointima formation (Figure 5C; represented by a green “+” icon). Despite the similar molecular structures of different IRF family members, it could also indicate that different IRFs employ different mechanisms by which they affect neointima formation (see above).

### 8.3. Modulation of Macrophage Differentiation: Anti-Atherogenic vs. Pro-Atherogenic

Also, multiple studies point to a crucial role of KLF4 in MØ differentiation upon inflammatory stimuli, acting as a transcriptional activator or repressor and demonstrating both pro- as well as anti-inflammatory properties. Studies by Liao et al. provided evidence for a differential effect of KLF4 on expression of genes that characterize the M1 and M2 phenotype [14,131,133]. With respect to M2 polarization in response to IL-4, KLF4 cooperated with STAT6 in a positive-feedback manner to increase expression of M2 anti-inflammatory genes (Figure 5A,D; represented by a green “+” icon). At the same time, KLF4 also inhibited the M1 pathway (Figure 5D; represented by a red “minus” icon) by interfering with NF-κB recruitment to pro-inflammatory gene promoters in response to IFNγ and LPS (Figure 5D) [131]. Likewise, KLF4 may also be important in the suppression of pro-inflammatory genes in M2 MØ. Mechanistically, KLF4 was shown to compete with key NF-κB coactivators such as p300/CBP and associated factors such as PCAF [131].

These anti-inflammatory characteristics of KLF4 were shown to be in line with a study in which KLF4 was identified as a negative regulator of the cellular antiviral immune response [148]. KLF4 overexpression inhibited SeV-mediated dose-dependent activation of IFN-β promoter and ISRE activity in a variety of cell types. Under similar conditions, knockdown of KLF4 increased ISG expression, including *IFNB1*, *RANTES*, and *ISG15*, and inhibited replication of VSV. Moreover, translocation of KLF4 from cytosol to nucleus was observed upon viral infection and concomitant inhibition of ISRE and NF-κB-mediated transcription. Moreover, ChIP analysis after viral infection confirmed recruitment of KLF4 to the *IFNB* gene promoter in a competitive fashion with IRF3. These results provide evidence to suggest that KLF4 plays an inhibitory role in virus-triggered type I IFN signaling by competing with the collaborative activity of NF-κB and IRF3 towards transcriptional regulation of *IFNB* and ISGs. Possibly, this could also account for other IRFs (IRF7, IRF5, IRF9) and STATs (STAT1 and STAT2), which have shown to play key roles in the transcriptional regulation of IFNs and ISGs and anti-viral response (Figure 5D; represented by a red “minus” icon) [55,148].

Similarly, in human diseased epidermal keratinocytes, KLF4 was shown to act as a negative regulator of IFNγ-induced transcriptional regulation of the *SOCS1* gene by competing with IRF1. Mechanistically, it was shown in healthy keratinocytes that KLF4 is involved not only in the repression of *SOCS1* in basal conditions but subsequently replaced by IRF1 upon IFNγ treatment, leading to increased *SOCS1* expression (Figure 5D; represented by a red “minus” icon). In psoriatic keratinocytes, the expression of KLF4 is significantly decreased, leading to an imbalance where the positive regulator IRF1 has a greater impact on *SOCS1* transcription. Thus, its overexpression contributes to the inflammatory response in psoriasis by dampening the effects of IFNγ [149].

Also, Langlais et al. identified IFNγ-induced co-binding of IRF1, IRF8, STAT1, and PU.1 to ISRE-containing genes in MØ directed expression of a set of genes, the IRF8/IRF1 regulome, that play critical roles in host inflammatory and antimicrobial defenses [73]. It is therefore tempting to speculate that under these conditions, KLF4 acts as a negative regulator of IFNγ-induced expression of inflammatory genes in MØ by modulating the DNA binding of IRF1, IRF8, and STAT1 (Figure 5D; represented by a red “minus” icon).

As mentioned above, under specific conditions and mechanisms such as SENP1-mediated de-SUMOylation of KLF4, KLF4 shifts its function to enhance MØ M1 polarization and promote atherosclerosis (Figure 4E; represented by a green “+” icon). Evidence suggests that this happens via NF-κB activation, leading to increased production of pro-inflammatory cytokines (Figure 5E; represented by a green “+” icon) [133]. Indeed, Feinberg et al. revealed in MØ in response to IFNγ, LPS, or TNF that KLF4 interacts with the NF-κB family member p65 (RelA) to cooperatively activate the *iNOS* promoter [137]. Likewise, KLF4 was shown to modulate expression of *IL-6* in dendritic cells and in fibroblast-like Synoviocytes in response to LPS or TNFα, through binding to the *IL-6* promoter and direct interaction with NF-κB [139,140]. In addition, it was found that KLF4 overexpression promoted the M1 polarization of RAW 264.7 cells, accompanied by increased STAT1 expression and activity. Moreover, KLF4 overexpression aggravated synovial tissue inflammation and injury in mouse joints in a STAT1-dependent manner [138]. Under these conditions, activation of STAT1, NF-κB, and different IRFs coordinates robust expression of multiple chemokines, adhesion molecules, and antiviral and antimicrobial proteins, including *iNOS* and *IL-6*, and predicts a pro-inflammatory collaboration with KLF4 (Figure 5E).

Finally, a promoting role of KLF4 was discovered in the inflammatory transcriptional program of IRF8-dependent monocyte/MØ differentiation. Kurotaki et al. highlight the essential role of the IRF8-KLF4 transcription factor cascade in the development of Ly6C+ monocytes from murine myeloid progenitors [150]. The research demonstrates that IRF8 induces the expression of *KLF4* (Figure 5A; represented by a green “+” icon), and this cascade is crucial for the proper differentiation of monocytes. Specifically, IRF8 binding to DNA, both at promoter-proximal and promoter-distal regions, is associated with gene induction, including *KLF4*, and is accompanied by increased histone H3K4me1, a marker of enhancers. This IRF8-KLF4 interaction is vital for the development of Ly6C+ monocytes, a specific subset of monocytes that plays important roles in host defense and inflammation.

## 9. Perspectives

The multi-functional transcriptional regulator and pluripotency factor KLF4 acts as a gatekeeper of VSMC phenotypic switching and MØ polarization during vascular inflammation and atherosclerosis. Likewise, pro-inflammatory pathways activated by TLR4 and IFNγ emerge as key components of VSMC and MØ plasticity, tightly regulated by STATs, IRFs, and NF-κB. Recent discoveries predict a collaborative role of these transcription factors in different transcriptional mechanisms connected to inflammation and atherosclerosis (Figure 5). These multiple mechanisms are driven by the dual regulatory role of Klf4 in vascular injury and inflammation and seem context- and cell-type dependent. In VSMCs, selective collaborations of KLF4, STAT, IRF, and NF-κB either promote VSMC phenotypic switching and atherogenesis or inhibit VSMC proliferation and protect against neointima formation (Figure 5B,C). In MØ, on the other hand, interplay of KLF4, STAT, IRF, and NF-κB has both pro- and anti-inflammatory outcomes connected to atherosclerosis (Figure 5D,E). Currently it is not clear how KLF4 switches between pro- and anti-atherogenic activity or between transcriptional activation and inhibition of target genes, depending on external factors. Likewise, it has not been clarified how this is effected by genome-wide interactions with specific STATs, IRFs, and NF-κB in relation to VSMC phenotypic switching and proliferation and MØ plasticity.

It is interesting to note that a number of studies support both a pro- and anti-tumorigenic role for KLF4 in cancer cells. The p21 status of cancer cells could be a potential explanation for this phenomenon. With the cell-type dependent roles in VSMCs and MØ, it could be suggested that the cellular context likely affects KLF4 action as well as its collaborative characteristics with specific STATs, IRFs, and NF-κB. It is therefore a main challenge to identify those conditions, and the effects on different cellular phenotypes, under which KLF4 acts as a repressor or as a transcriptional activator in collaboration with different STATs, IRFs, and/or NF-κB, and how this effects vascular function during atherosclerosis.

The advent of scRNAseq and spatial transcriptomics has enabled the study of gene expression and regulation in disease and development at the single-cell level. By exploring circulating blood cells or predefined markers applied to the human artery, the cellular composition in atherosclerosis has previously been studied [151]. Recent scRNAseq and spatial transcriptomics studies on human and mouse atherosclerotic tissue have discovered new insight at the cellular resolution level. This has provided more detailed information on plasticity, heterogeneity and the involvement of different cell types in atherogenesis [38,152]. As a first, in 2024, Bleckwhel et al. generated a novel integrative high-resolution map of human coronary/carotid atherosclerotic plaques by combining new spatial transcriptomics data from 12 human specimens with scRNAseq datasets from previously published studies. Integrative analyses observed cell-type and atherosclerosis-specific expression differences in association with changes in cell–cell communication and improved our understanding of disease mechanisms. In combination with an existing drug database, the potential of this novel atlas was also tested for identifying novel drug targets with cell state and spatial niche specificity [153]. These advanced technologies raise the possibility for studying in more detail the molecular networks of KLF4, STATs, IRFs, and NF-κB in VSMC and MØ subtypes and their cellular interplay in the dynamic atherosclerotic plaques and offer novel mechanistic insight. This could also lead to the identification of novel VSMC and/or MØ-specific clinical biomarkers and the development of novel diagnostic tools to monitor and diagnose plaque-specific inflammatory responses in a cell-type dependent manner. Moreover, given its cell-type and context-dependent nature, targeting these multiple transcriptional collaborations presents an interesting, yet complex, candidate for tailored, targeted therapeutic intervention in atherosclerosis. Inhibiting selective KLF4-, STAT-, IRF-, and NF-κB-dependent transcriptional activities in VSMCs could restore contractility and abrogate pro-inflammatory responses to encumber atherosclerotic progression. In contrast, enhancing or blocking activity of specific KLF4, STAT, IRF, and NF-κB collaborations in MØ may promote anti-inflammatory MØ polarization to prevent tissue damage and inhibit atherogenesis. Combined with our newly developed selection strategy for STAT and IRF inhibitors [4,41,88,154], this could lead to the development of VSMC- and MØ-targeted therapies in the battle against atherosclerotic disease.

## Data Availability

No new data were created or analyzed in this study. Data sharing is not applicable to this article.

## Abbreviations

The following abbreviations are used in this manuscript:

EC	endothelial cells
TLR	Toll-like receptors
MØ	macrophages
Th1	T helper 1 lymphocytes
IFN	interferon
DC	dendritic cells
VSMC	vascular smooth muscle cells
KLF4	Krüppel-like factor 4
STAT	Signal Transducer and Activator of Transcription
IRF	Interferon Regulatory Factor
NF-κB	Nuclear factor-κB
SM-MHC/MYH11	smooth muscle myosin heavy chain
CNN1	calponin
SM22α	transgelin
MYOCD	myocardin
TNF-a	tumor necrosis factor alpha
PDGF-BB	platelet-derived growth factor BB
ROS	reactive oxygen species
LPS	lipopolysaccharide
NO	nitric oxide
ApoEKO	Apolipoprotein E Knockout
LDL	low-density lipoprotein
LDLr	low-density lipoprotein receptor
WT	wild-type
HFD	high fat diet
GAF	γ-activated factor
GAS	IFNγ-activated sequence
ISGF3	interferon-stimulated gene factor 3
ISRE	IFN-stimulated response element
CVD	cardiovascular disease
Dnmt3a	DNA methyltransferase 3a
DBD	DNA-binding domain
TAD	Transcriptional activation domain
iNOS	Inducible Nitric Oxide Synthase
MSC	mesenchymal stem cells
SUMO	small ubiquitin-like modifier
SENP1	small ubiquitin-like modifier specific peptidase 1
TG	transgenic
CKD	chronic kidney disease
scRNAseq	single-cell RNA sequencing
